# Personal Health Record Use in the United States: Forecasting Future Adoption Levels

**DOI:** 10.2196/jmir.4973

**Published:** 2016-03-30

**Authors:** Eric W Ford, Bradford W Hesse, Timothy R Huerta

**Affiliations:** ^1^ Department of Health Policy and Management Johns Hopkins University Baltimore, MD United States; ^2^ National Cancer Institute Bethesda, MD United States; ^3^ College of Medicine Departments of Family Medicine and Biomedical Informatics The Ohio State University Columbus, OH United States

**Keywords:** personal health records, electronic health records, patient participation, technology diffusion, Bass modeling, PHR Adoption Forecasts

## Abstract

**Background:**

Personal health records (PHRs) offer a tremendous opportunity to generate consumer support in pursing the triple aim of reducing costs, increasing access, and improving care quality. Moreover, surveys in the United States indicate that consumers want Web-based access to their medical records. However, concerns that consumers’ low health information literacy levels and physicians’ resistance to sharing notes will limit PHRs’ utility to a relatively small portion of the population have reduced both the product innovation and policy imperatives.

**Objective:**

The purpose of our study was 3-fold: first, to report on US consumers’ current level of PHR activity; second, to describe the roles of imitation and innovation influence factors in determining PHR adoption rates; and third, to forecast future PHR diffusion uptake among US consumers under 3 scenarios.

**Methods:**

We used secondary data from the Health Information National Trends Survey (HINTS) of US citizens for the survey years 2008, 2011, and 2013. Applying technology diffusion theory and Bass modeling, we evaluated 3 future PHR adoption scenarios by varying the introduction dates.

**Results:**

All models displayed the characteristic diffusion S-curve indicating that the PHR technology is likely to achieve significant market penetration ahead of meaningful use goals. The best-performing model indicates that PHR adoption will exceed 75% by 2020. Therefore, the meaningful use program targets for PHR adoption are below the rates likely to occur without an intervention.

**Conclusions:**

The promise of improved care quality and cost savings through better consumer engagement prompted the US Institute of Medicine to call for universal PHR adoption in 1999. The PHR products available as of 2014 are likely to meet and exceed meaningful use stage 3 targets before 2020 without any incentive. Therefore, more ambitious uptake and functionality availability should be incorporated into future goals.

## Introduction

The 2009 US Health Information Technology for Economic and Clinical Health (HITECH) Act called for the creation of a meaningful use (MU) incentive program to distribute significant financial support to providers and health systems adopting electronic health record (EHR) technologies [1,2]. The program has been successful in boosting EHR adoption rates at least in the short term by applying exogenous incentives to a market that had otherwise been stalled. This was considered by many policy makers to be a necessary first step in establishing the infrastructure that could eventually be leveraged to improve the quality of care delivery and to encourage patients’ engagement in improving their own health outcomes. In an external review led by the Robert Wood Johnson Foundation [3], reviewers observed that while the overall impact of HITECH and its many programs may not yet be clear, “the pace of adoption of technologies by the public is likely to continue at a rapid pace.” Furthermore, the reviewers concluded, “Consumer engagement with technology is likely to bring further pressure to bear on health care organizations as patients seek ways to use these devices to track and transmit their own data and interact with health care health professionals.”

This notion that consumer involvement would be a key to the success of HITECH in bringing about improved health outcomes was central to the formulation of the MU incentive program. In a report by the National Research Council (National Academies of Sciences, Engineering, and Medicine of the United States) released in 2009, a task force of informatics scientists noted that, for computational technology to be effective in health care improvement, it must provide functionality and cognitive support that is of value to providers, patients, and their caregivers [4]. Stage 2 of the MU incentive program requires the active engagement of patients and their families with patient portal technologies in managing their own health information and care coordination [5-7]. Stage 3 MU recommendations (originally scheduled for implementation in 2017 but now under policy reconsideration) state that patients should be able to (1) communicate electronically using secure messaging, (2) access patient education materials on the Internet, (3) generate health data into their providers’ EHRs, and (4) view, download, and transmit their provider-managed EHRs. Taken together, these requirements outline the basic functionalities of a consumer-managed personal health record (PHR) [8].

PHRs offer a tremendous opportunity to generate consumer support in pursuing the triple aim of reducing costs, improving health outcomes for populations, and improving the experience of care for patients and their families [9-13]. Moreover, surveys have indicated that consumers want Web-based access to their medical records [14,15]. Nevertheless, diffusion of full patient access to their EHR-tethered portal or personally controlled PHR has been slow historically. Reasons given have included worries on the patients’ side that full and open access to personal medical information could bring up privacy concerns; worries on the providers’ side that the technical nature of Web-based medical information could create a health literacy burden; and concerns on the business side that MU stage 2-certified EHRs are not set up to support fully interoperable data exchange [16]. These concerns notwithstanding, a set of converging trends may be pushing consumer access to PHR functionality toward a patient-driven health information economy. Nearly two-thirds of the American public own mobile phones and have become accustomed to interactive services related to personal data in other facets of their lives. As reimbursement models change, health care providers will need to incorporate data from multiple sources in order to get a better picture of the total patient’s preventive health needs [17]. For these reasons, there is an emerging need for more research into consumer engagement [9,14]. Given the current state of consumer usage levels, and observations associated with the diffusion of innovations in other settings, it should be possible to forecast PHR adoption uptake and explore how imitation and innovation factors are influencing the pattern.

The purpose of our study was to estimate the future uptake of PHR functionalities among the US population. We analyzed the Health Information National Trends Survey (HINTS), a nationally representative survey conducted by the US National Cancer Institute, to assess consumers’ current use of Web-based apps to store personal health information and communicate with providers [18]. We also used the data to forecast the future adoption of these PHR apps.

Understanding the trajectory of PHR uptake by consumers is important for policy makers, providers, and technology vendors. For policy makers, setting PHR usage targets based on quantified estimates rather than normative goals will ensure that targets are set at optimal levels to accelerate uptake, but not be unachievable. The provider community has been resistant to health information sharing. Having evidence that consumers are not only willing, but also able, to effectively use such tools may lower this resistance. In addition, having an active and growing market for PHR technologies should spur health information technology vendors to invest in research and development to take advantage of this burgeoning market.

## Methods

### Data Source, Variables, and Sample

We analyzed 3 iterations of the HINTS version 4 survey of US adults (survey years 2008, 2011, and 2013). A calculated variable based on survey responses to 2 questions measured PHR functionality: (1) “In the last 12 months, have you used the Internet to keep track of personal health information such as care received, test results, or upcoming medical appointments?” and (2) “In the last 12 months, have you used email or the Internet to communicate with a doctor or a doctor’s office?” These items capture 2 critical requirements for effective use of a PHR: storing clinical data electronically and communicating with a care provider over the Internet. We considered respondents answering yes to both items to be using basic PHR functionalities as described in the Centers for Medicare & Medicaid Services’ MU program for EHRs. Data were weighted according to specifications provided by the National Cancer Institute to make the data representative of the United States overall. Adoption rates were calculated for use in the Bass model analysis.

### Bass Modeling and the Technology Diffusion Model

Rogers [19] developed the technology diffusion theory that describes how innovators’ (ie, first adopters), early adopters’, early majority, late majority, and laggards’ adoption patterns occur over time. Subsequently, Bass [20] developed the first commercial applications of such diffusion models, predicting the uptake of consumer products based on the influence of various types of advertising campaigns and motivations internal to the customer. The Bass model predicts how many customers will eventually adopt a new product, and when they will do so, based on early market penetration rates.

The model has several attractive properties. Empirical research by Bass [20] identified the latent factors that predict a technology’s diffusion pattern as a function of external and internal influences. The *external influence coefficient*, represented by the letter p in the empirical model, represents the impact of innovation and advertising and the environmental context in which the innovation is embedded. In contrast, the *internal influence coefficient*, represented by the letter q, reflects the impact of relationships on diffusion, and is therefore often referred to as the imitation coefficient, the word-of-mouth effect, or social contagions in the diffusion literature [21].

The parameters *p* and *q* provide information about how a new technology will diffuse in the future. A high external influence coefficient (*p*) indicates that the diffusion has a quick start but also tapers off quickly. A high internal influence coefficient (*q*) indicates that the diffusion starts slowly and accelerates later as the product’s benefits are spread, typically by word-of-mouth.

In concert, these variables interact to create diffusion dynamics. For example, when the internal influence coefficient (*q*) is larger than external influence coefficient (*p*), the cumulative number of adopters follows the type of S-curve often observed for high-risk, innovative products that take extended time frames to become widely used. When the internal influence coefficient (*q*) is smaller than the external influence coefficient (*p*), the cumulative number of adopters follows an inverse J-curve trajectory, often observed in less-risky innovations, such as new consumer durables (eg, washers and dryers).

### Analysis

We conducted a sensitivity analysis to test the Bass model’s parameters and create a possible range of future PHR uptake by varying the technology introduction year [22]. The oldest estimates for PHRs entering the market place with the minimum functionalities described above puts their introduction around the year 2001 [23]. Halamka et al [24] documented the developmental period for the first PHRs and identified 2007 as being the first year that clinically based apps accessed through providers’ systems, rather than Web-based technologies managed by consumers (eg, HealthVault and WebMD), were widely available to the public. Similarly, Kaiser Permanente, a US managed care provider, made its clinically linked PHR available to all members in 2007 [25]. Therefore, we used 2001 and 2007 as potential PHR innovation start dates. Additionally, we analyzed 2004 as a midpoint to assess model fit.

We conducted the statistical analyses and forecasts using linear optimization in Microsoft Excel for Mac 2011 (Microsoft Corporation). The model was constrained to ensure that the theoretical model fit within 2% of actual data throughout the estimates. The models were analyzed using both generalized reduced gradient algorithm for nonlinear functions and the evolutionary algorithm for assessing discontinuous change. The generalized reduced gradient algorithm identified better model fits in every instance and they are the only results we report for this study. As an additional model reliability assessment, we reran the models with their last year of data omitted to assess how the trends would vary under different amounts of input.

## Results

Over the survey years, consumers were increasingly using electronic media for both storing health data and communicating with their clinical providers (see Table 1). Based on survey weighting, approximately 8 million people were using the 2 basic PHR functionalities tracked in 2008 (eg, storing data on the Internet and communicating electronically with a clinical provider). Similar to other Internet-based social media, the PHR functionality uptake among consumers grew rapidly and exceeded 31 million users in 2013 [26].

In addition to the rapid growth in the number of individuals using the two technologies that are at the core of the PHR together, there was a steady growth in the number of consumers using one of the tools to manage the flow of health information. In particular, the use of technology to communicate directly with clinicians has been growing rapidly.

The PHR adoption scenario that used a 2001 technology introduction date had the tightest constraints and generated estimates that most closely approximated the observed experiences to date (see Table 2). The 2004 start date performed next best, with the 2008 and 2013 estimates slightly understating the observed rates of PHR use. The 2007 start date’s estimates performed in a similar pattern to the 2004 version, albeit in a slightly more exaggerated fashion. These differences are caused by the internal and external coefficients that underlie the models’ operations moving to more extreme values.

The external (*p*) and internal (*q*) coefficients for the 2001 and 2004 PHR introduction dates are consistent with results from a wide range of other products’ diffusion patterns that have been studied using the Bass model [27]. The sensitivity analysis shows that the PHR diffusion models with a 2004 start date provide a motivation coefficient ratio (*q*/*p*=30.092) that is the most similar to prior studies from other domains. Assessing the 2004 model’s stability by omitting the last year’s data did not significantly change the forecasts, and the estimate for year 2020 was within seven-tenths of 1% of the model using data from the 3 years 2008, 2011, and 2013.

The internal coefficient (*q*) for 2007 is lower than in most prior studies but is still plausible. The external coefficient (*p*) for the 2007 start date is within the normal range (see Table 3). Moreover, all 3 Bass models suggest that PHR-like, Internet-based personal health information management innovations will make significant gains in future.

**Table 1:** Extrapolated response rates for items of interest measuring PHR^a^ functionality based on HINTS^b^ weightings.

Responses to items^c^	Survey year		
	2008	2011	2013
Yes to both PHR items, n (%)	7,878,118 (5.16%)	15,407,840 (9.80%)	31,220,465 9 (17.17%)
Yes to clinician communication item only, n (%)	12,881,980 (8.44%)	14,665,440 (9.32%)	22,880,580 (12.58%)
Yes to tracked personal health information item only, n (%)	13,897,188 (9.11%)	14,761,217 (9.39%)	19,969,109 (10.98%)
No to both PHR items, n (%)	117,944,796 (77.29%)	112,444,964 (71.49%)	107,794,014 (59.27%)
Total number of responses	152,602,082	157,279,461	181,864,168

^a^PHR: personal health record.

^b^HINTS: Health Information National Trends Survey. Data are reweighted to create a nationally representative sample.

^c^Questions regarded whether respondents used (1) Internet-based health information storage and (2) Internet-based communication with physicians in the past year.

**Table 2:** Differences between HINTS^a^ survey results and Bass modeling estimates for personal health record adoption among US consumers.

		Observed uptake rate (survey results)	Technology introduction start date					
			2001		2004		2007	
			Bass	Difference	Bass	Difference	Bass	Difference
HINTS year								
	2008	5.16%	4.54%	–0.623	4.36%	–0.800	3.96%	–1.20
	2011	9.80%	10.50%	0.700	10.60%	0.800	11.00%	1.20
	2013	17.17%	17.17%	0.000	16.82%	–0.352	16.41%	–0.76
Mean difference				0.020		0.117		0.253

^a^Health Information National Trends Survey (HINTS) for 2008, 2011, and 2013 serve as the known observations. Bass estimates are based on the first year when various experts identify a minimally functioning personal health record being available in the marketplace.

**Table 3:** Sensitivity analyses for internal and external coefficients.

		External coefficient (*p*)	Internal coefficient (*q*)	Motivation coefficient ratio (*q*/*p*)
Innovation introduction start date				
	2001	0.002	0.268	117.040
	2004	0.007	0.214	30.092
	2007	0.018	0.095	5.181
MU^a^ targets		0.002	0.217	148.44

^a^MU: meaningful use incentive program of the US Health Information Technology for Economic and Clinical Health (HITECH) Act. The start date for personal health record availability for the MU model is 2004.

The MU model estimates a diffusion curve based on personal health record adoption targets set by policy. The resultant curve suggests the diffusion of these innovations if we assume the MU minimum targets for the diffusion trajectory. Notably, the external coefficient (*p*=.002) of the MU policy targets creates an expectation of diffusion that is very low compared with most other research on technology adoption. We found that MU policy targets create a diffusion pattern similar to what we might find if we had started this policy in 2001 with a much lower social contagion effect (*q*/*p*=148.44). The net result for the MU stage 2 and 3 targets is a diffusion pattern of much lower consumer uptake of PHR functionalities than would occur under conditions without an intervention.

**Figure 1:**
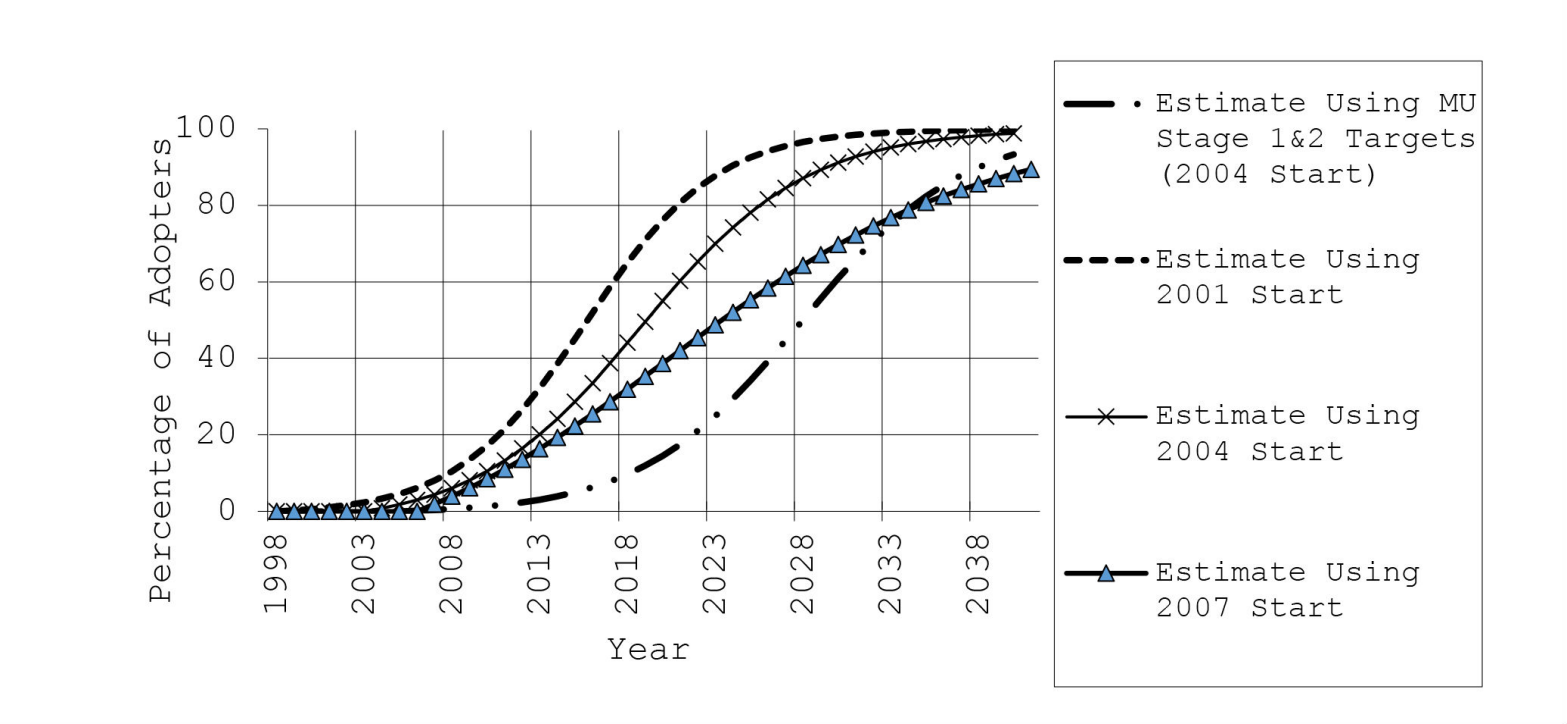
Projected diffusion patterns for Internet-based personal health record adoption in the United States, by year of introduction. MU: meaningful use incentive program of the US Health Information Technology for Economic and Clinical Health (HITECH) Act.

Graphing the PHR diffusion models, all the versions display the characteristic S-curve of a product adoption that will be sustainable (see Figure 1). Both the 2001 and 2004 start-date forecasts indicate that a plurality of the US population will be using key PHR functionalities by 2020 in excess of the MU goals. Only the 2007 start-date curve indicates that there will be a diffusion rate that will not meet most proposed MU program objectives for PHR adoption.

## Discussion

The diffusion of PHR-like apps among US consumers is proceeding rapidly. Based on the high values for the internal coefficient (*q*) on the best-performing model (ie, 2004 PHR introduction date), the diffusion rate is following the trajectory associated with the long-term adoption of consumer-driven technologies. Moreover, the adoption trajectories for all of the observed models exceed the policy targets articulated for MU stages 2 and 3.

With respect to PHR policy incentives, the MU program has included inducements for health systems and providers to make PHR functionalities available to consumers [28]. MU stage 2 requires EHR systems to allow patients to see their medical records, transmit their records to others, and communicate with their provider through a secure portal. Specifically, 5% of patients must be using the provider communication functionality by 2014 for the provider to be eligible for reward payments. These are minimal capability and engagement targets, and no specific populations are identified as being most likely to benefit [29,30]. As described in the regulation, the record sharing does not require an interoperable record that would allow another provider to bring data into its own system in a structured fashion. This results in data exchange with limited utility for patients using a third-party’s or other provider’s PHR app.

MU stage 3 has slightly higher thresholds for consumer engagement than stage 2. The percentage of consumers who must communicate electronically with their provider rises from 5% under stage 2 (target date of 2014) to 10% under stage 3 (target date of 2017). The percentage of consumers who must have access to their entire record under stage 3 targets is 50%. Neither stage 2 nor stage 3 requires the downloaded PHR be interoperable with other providers’ health information technology systems.

The MU model’s slower consumer adoption rate is driven by a low external coefficient (*p*=.002) relative to the 2004 technology introduction forecasts (*p*=.007). The low external coefficient suggests that the MU program may be having the opposite of its intended effect by slowing innovation. This is likely explained by 2 factors. First, health information technology system vendors and providers may be making the minimum PHR functionalities available instead of adopting the higher-level capabilities available in nonclinical contexts that attract consumers to engage with these tools. For example, the user experience of some personal health tracking tools is much better and stylized than the current PHR tools. While numerous groups, as well as the US government, have explored potential design options for improving the user experience in this context, those innovations have not found their way into the current generation of PHRs.

Second, care providers may be creating barriers to technology adoption through bureaucratic and administrative burden, although most have attested to exceeding the MU standards [31]. There is a significant concern about the liability of releasing PHR data outside the institutional firewall. The result is significant adoption costs, especially in terms of time, for patients using PHRs. The internal coefficient (*q*=.217) for the MU program forecast is comparable with the other models, suggesting that, under any circumstance, consumer demand for PHR functions will remain strong. Using the stage 2 and 3 consumer engagement targets as diffusion forecasts yields the lowest consumer uptake rates of any model. The internal to external coefficient ratio (*q/p* = 148.44) is the highest of any forecast, suggesting that consumers’ desire for the product, rather than ongoing product innovations, will be the primary driver of PHR diffusion rates under this scenario.

### Limitations

The research described herein has 3 main limitations. First, the HINTS instrument provides valuable insights into consumer behavior; however, the questions asked in earlier iterations did not explicitly refer to PHR technologies. Therefore, the results are only an approximation of the actual phenomenon. Second, a limitation inherent in the HINTS instrument is that it does not account for other people managing someone else’s health information. In many households, one individual manages the care for other family members, including children and parents. The extent to which this is being carried out electronically was not measured. Thus, the actual prevalence of PHR use may be higher. Third, it is likely that new PHR functionalities will fundamentally change the technology and be, in effect, a new product. The release of a “new” product versus an “updated” iteration means there will be a new start date for the PHR introduction, which will change the curve profiles. A better understanding of what constitutes a new product in this marketplace merits discussion.

### Conclusion

Consumers’ PHR use is growing in both the numbers of people engaged and the degree of technological functionality they can manage [32]. As organizations identify ways to make these tools more widely available, sophisticated PHR technologies would move from the domain of early adopters to the widespread use among a majority of consumers in the market. As this occurs, the primary factor limiting PHR functionalities’ diffusion may well be health care vendors’ and providers’ reticence to deploy these tools in a manner that resonates with the patient. It is *not* the consumer who is unwilling to use these tools, but the deployment and barriers they face that limits their adoption.

Vendors and providers are not the only component slowing adoption. MU goals on this issue may have the same problems in PHR adoption that they experienced in EHR adoption: the standards of engagement are low enough to allow for incremental approaches to adoption as opposed to incentivizing transformative targets. Policy discussions in a “post-meaningful use” world would benefit from insights provided through these types of data-based diffusion analyses, especially as the emphasis shifts away from applying endogenous incentives for adoption, to driving innovation to curry the interest of engaged consumers [33]. Robert Wachter, in his book *The Digital Doctor: Hope, Hype, and Harm at the Dawn of Medicine’s Computer Age*, summed up the future prospects of health information technology this way: “The real action—and the money—will shift to creating innovative tools to allow patients to stay healthy and to manage chronic illness” [34].

## Abbreviations

EHR: electronic health record
HINTS: Health Information National Trends Survey
HITECH: Health Information Technology for Economic and Clinical Health
MU: meaningful use
p: external influence coefficient
q: internal influence coefficient
PHR: personal health record
